# Circulating tumour cells for guiding antibody‒drug conjugate therapy: Role of artificial intelligence

**DOI:** 10.1002/ctm2.70748

**Published:** 2026-09-24

**Authors:** Vahid Yaghoubi Naei, Mehran Dabiri, Lihua Chen, Jiajia Li, I‐Han Wang, Gungun Lin, Majid Ebrahimi Warkiani

**Affiliations:** ^1^ Institute for Biomedical Materials & Devices School of Mathematical and Physical Sciences Faculty of Science University of Technology Sydney Sydney New South Wales Australia; ^2^ School of Biomedical Engineering University of Technology Sydney Sydney New South Wales Australia; ^3^ Department of Gynecologic Oncology Fudan University Shanghai Cancer Center Fudan University Shanghai China; ^4^ Department of Clinical Medicine Suzhou Labyrinth Biotech Co., Ltd Suzhou China; ^5^ Department of Mechanical Engineering College of Engineering American University of Sharjah Sharjah United Arab Emirates; ^6^ Centre for Biomedical Innovations (CBI) American University of Sharjah Sharjah United Arab Emirates

**Keywords:** antibody‒drug conjugates, artificial intelligence, circulating tumour cells, epithelial‒mesenchymal transition, liquid biopsy, precision oncology, tumour heterogeneity

## Abstract

Antibody‒drug conjugates (ADCs) represent a major advance in precision oncology, yet their effectiveness depends critically on adequate target antigen expression. Tumour heterogeneity and dynamic antigen expression during treatment pose major challenges for conventional tissue biopsies used in patient selection. Liquid biopsy components such as circulating tumour cells (CTCs) enable real‐time, minimally invasive monitoring of tumour burden, molecular characteristics and target antigen status. This review evaluates the potential of CTC analysis to overcome the key challenges in ADC therapy, including real‐time target profiling, enhanced patient stratification and longitudinal monitoring of treatment response. While acknowledging that direct clinical evidence for CTC‐guided ADC selection remains limited and prospective validation is still needed, we integrate clinical evidence from CTC‐guided trials across different cancer types and propose pragmatic decision‐making frameworks to incorporate CTC testing into ADC treatment strategies. We also examine how artificial intelligence (AI) has improved CTC detection accuracy and discuss exploratory AI models for multi‐omics integration and hypothetical AI‐guided ADC recommendation systems, clearly distinguishing these by their current level of clinical evidence. Liquid biopsy‐guided ADC selection has the potential to enable more personalised cancer treatment as CTC technologies advance and standardisation improves.

## Graphical abstract

This schematic demonstrates how tissue and liquid biopsies facilitate ADC target selection. (A) Demonstrating the tumour microenvironment and the systemic dissemination of CTCs. The primary tumour, nesting a complex microenvironment of immune and stromal cells, shed tumour cells into the bloodstream, facilitating haematogenous spread. CTCs have clinically useful surface antigens on them, such as human epidermal growth factor receptor 2 (HER2), trophoblast cell surface antigen 2 (TROP2), Nectin‐4, B7‐H3, CDH6, tissue factor (TF), folate receptor alpha (FRα) and NaPi2b. These are either well‐known or new targets for approved ADCs, highlighting the biological link between tumour heterogeneity, vascular dissemination and targetable ADC biomarkers in solid cancers. (B) This comparison highlights tissue and liquid biopsies abilities for tumour analysis and treatment tracking. Tissue biopsy offers localised, invasive and static information, which limits its ability to detect intratumoural heterogeneity, early disease progression and long‐term monitoring. In contrast, liquid biopsy enables minimally invasive and repeatable sampling of circulating tumour components, providing a better understanding of tumour heterogeneity. This increases the chances of early detection and allows real‐time tracking of disease dynamics during treatment. (C) Longitudinal CTC analysis helps to dynamically profile target antigens, molecular features and treatment‐induced changes over the treatment course. AI‐based frameworks integrate CTC data from multiple sources, including expression patterns, genetic alterations and molecular signatures. This helps select the optimal ADC, track treatment response in real time, and identify resistance early. By offering an adaptive, feedback‐driven process, it allows for iterative therapy adjustments, potentially enhancing treatment precision and overall clinical outcomes. Created by BioRender.

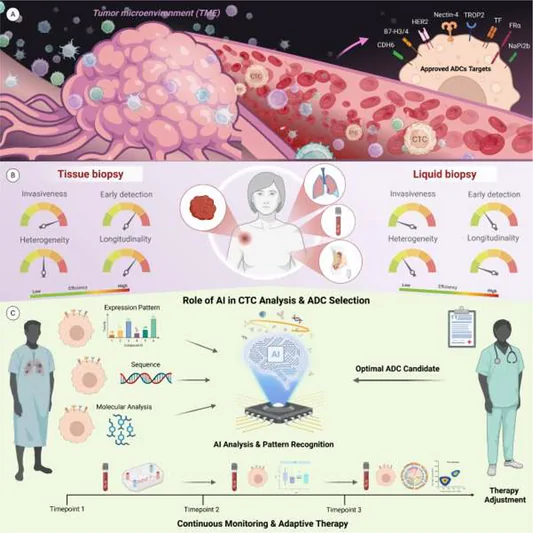

## 1 INTRODUCTION: THE NEED FOR DYNAMIC BIOMARKERS IN ADC THERAPY

Cancer continues to be one of the main causes of death worldwide, with metastasis being the key factor in cancer‐related mortality.^1^, ^2^ Antibody‒drug conjugates (ADCs) have emerged as one of the most promising developments in precision oncology, combining the targeting specificity of monoclonal antibodies with the cell‐killing potency of cytotoxic payloads to enable selective tumour‐directed delivery while limiting systemic toxicity.^3^, ^4^, ^5^, ^6^ Currently, 15 ADCs have been approved by the Food and Drug Administration (FDA) (based on Drugs@FDA database), and over 100 candidates are in clinical trials, underscoring the significant therapeutic potential of this treatment modality.^7^, ^8^, ^9^

**FIGURE 1:**
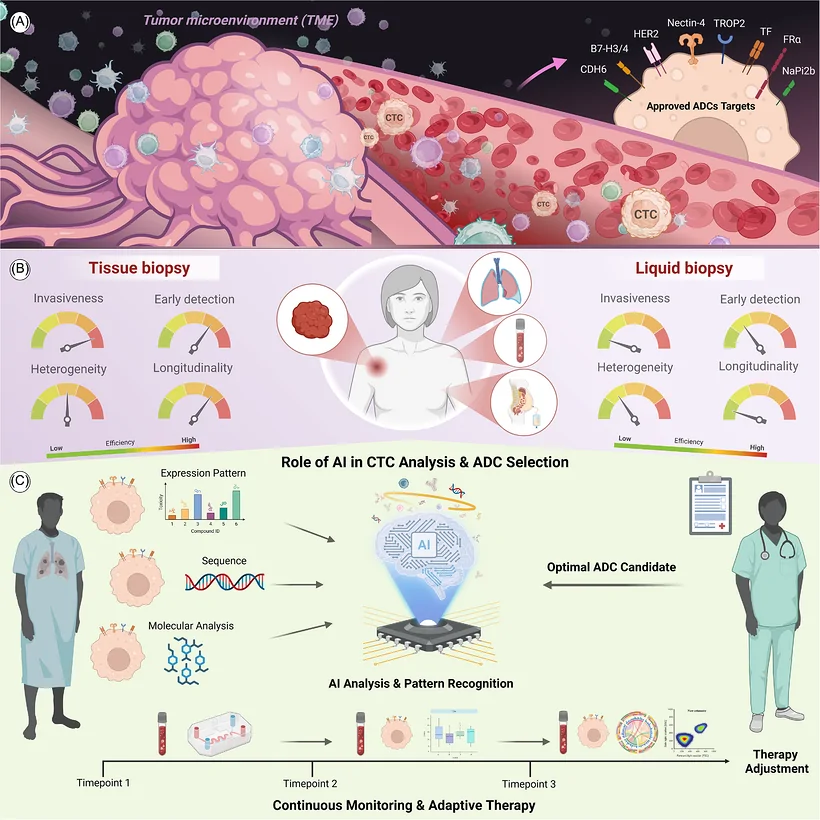
Schematic demonstrates how tissue and liquid biopsies facilitate antibody‒drug conjugate (ADC) target selection. (A) Demonstrating the tumour microenvironment (TME) and the systemic dissemination of circulating tumour cells (CTCs). The primary tumour, nesting a complex microenvironment of immune and stromal cells, shed tumour cells into the bloodstream, facilitating hematogenous spread. CTCs have clinically useful surface antigens on them, such as human epidermal growth factor receptor 2 (HER2), trophoblast cell surface antigen 2 (TROP2), Nectin‐4, B7‐H3, CDH6, tissue factor (TF), folate receptor alpha (FRα) and NaPi2b. These are either known or new targets for approved ADCs highlighting the biological link between tumour heterogeneity, vascular dissemination and targetable ADC biomarkers in solid cancers. (B) This comparison emphasises the potential of tissue and liquid biopsies for tumour analysis and tracking of treatment. Tissue biopsy is localised, invasive and static, limiting the detection of intratumoural heterogeneity, early disease progression and long‐term monitoring. On the other hand, liquid biopsy offers a minimally invasive and repeatable sampling of circulating tumour components and a better understanding of tumour heterogeneity. This can increase the likelihood of early detection and allows for real‐time tracking of disease dynamics during treatment. (C) Longitudinal CTC analysis allows dynamic profiling of target antigens, molecular features and treatment‐induced changes during the course of treatment. CTC data from different sources such as expression patterns, genetic alterations and molecular signatures are integrated in artificial intelligence (AI)‐based frameworks. This enables the choice of the best ADC, monitoring of treatment response in real time, and early detection of resistance. It provides a feedback‐based adaptive process that can be employed for iterative adjustments to therapy, potentially increasing precision and overall clinical benefits. Created by BioRender.

ADC efficacy depends critically on adequate expression of the target antigen at the tumour cell surface, a requirement complicated by the tumour microenvironment (TME), a dynamic ecosystem of cancer cells, stromal elements, vasculature and immune infiltrates exhibiting substantial spatial and temporal variation in antigen expression.^10^, ^11^, ^12^, ^13^ Antigen downregulation, inter‐regional expression heterogeneity and clonal evolution during treatment can all drive ADC resistance and treatment failure.^14^, ^15^ Although conventional tissue biopsies remain the gold standard for tumour diagnosis, they do not capture the dynamic complexity of tumours. They only provide a static snapshot of one region of one tumour at a single time point, which often does not reflect the overall molecular landscape of the disease or the changes in tumour cellular and molecular characteristics that occur during therapy (Figure 1B).^16^, ^17^

Given the limitations of tissue biopsies, there is increased interest in liquid biopsy components, especially circulating tumour cells (CTCs).^18^, ^19^ Among liquid biopsy components, both CTCs and circulating tumour DNA (ctDNA) have been investigated as complementary, minimally invasive alternatives to tissue biopsy, although each captures distinct biological information. In a direct head‐to‐head comparison in metastatic breast cancer, ctDNA showed a greater dynamic range and stronger correlation with radiographic tumour burden than CTC enumeration, providing an earlier readout of treatment response.^20^ However, because ADC efficacy depends on the expression, density and distribution of cell surface protein antigens, information that DNA‐based assays cannot directly capture, CTCs retain a distinct advantage for ADC‐specific applications: as intact cells, they allow direct single‐cell measurement of target protein expression alongside genomic and transcriptomic features. In this review, therefore, we focus on CTCs as the liquid biopsy component most directly amenable to antigen‐based ADC patient selection, while acknowledging ctDNA as a complementary tool, particularly for monitoring genomic resistance mutations. We do not argue that CTCs are the primary or only companion diagnostic approach to ADC therapy, but rather this review focuses on their specific and additive role in an ever‐evolving, multimodal liquid biopsy space involving ctDNA and tissue‐based testing.

CTCs are cancer cells that detach from primary or metastatic tumours and shed into the bloodstream, carrying molecular signatures reflective of both the primary tumour and its microenvironment (Figure 1A). Assessing CTCs offers a minimally invasive method for real‐time monitoring of the TME.^21^, ^22^ CTC analysis enables continuous tracking of tumour progression, revealing changes in antigen expression, genetic mutations and molecular responses to treatment that can assist in making more precise therapeutic decisions.^23^, ^24^ Especially in ADC therapy, real‐time monitoring of target antigen expression on CTCs can help select appropriate drugs, predict patient responses, identify resistance mechanisms and guide subsequent treatment plans; however, antigen expression alone reflects target availability rather than the full determinants of ADC sensitivity and prospective clinical validation in ADC‐treated cohorts remains limited.^25^, ^26^

Despite the great clinical potential of CTC‐based biomarkers, there are a number of key technical hurdles for their broad adoption, including the extremely low frequency of CTCs in peripheral blood, their phenotypic heterogeneity and the lack of standardised detection and analysis methods.^27^, ^28^ The rise of AI and machine learning (ML) offers potential solutions to these issues.^29^, ^30^ AI‐driven techniques have demonstrated clinical‐grade performance in standardising CTC detection through automated image analysis, while their use for managing complex multi‐omics data and creating predictive models for therapy response remains largely exploratory and has not yet been validated in prospective ADC‐treated cohorts.^31^, ^32^ ML algorithms can combine various data types, including antigen levels, genomic alterations and clinical information, to generate actionable insights for personalised treatment strategies (Figure 1C).^33^, ^34^

This review summarises current knowledge on the changing role of CTCs as dynamic biomarkers with the potential to guide ADC therapy in precision oncology. It addresses the potential role of CTC analysis for the management of tumour heterogeneity for ADC selection, therapeutic monitoring and prognosis and describes the potential of AI and ML to overcome technical challenges. By combining liquid biopsy techniques with advanced computational methods, this approach aims to enhance personalised treatment and improve outcomes for cancer patients receiving ADC therapies. Importantly, while CTC enumeration has prognostic value and CTC‐guided non‐ADC‐targeted therapy has been shown in prospective trials, direct evidence for CTC‐guided ADC selection and treatment sequencing is limited. The framework proposed here is thus meant to collate existing evidence and underscore a strategy that is promising from a clinical perspective and deserves dedicated prospective validation.

## 2 CTCS IN ADC SELECTION: ADDRESSING HETEROGENEITY AND DRUG TARGETING

**FIGURE 2:**
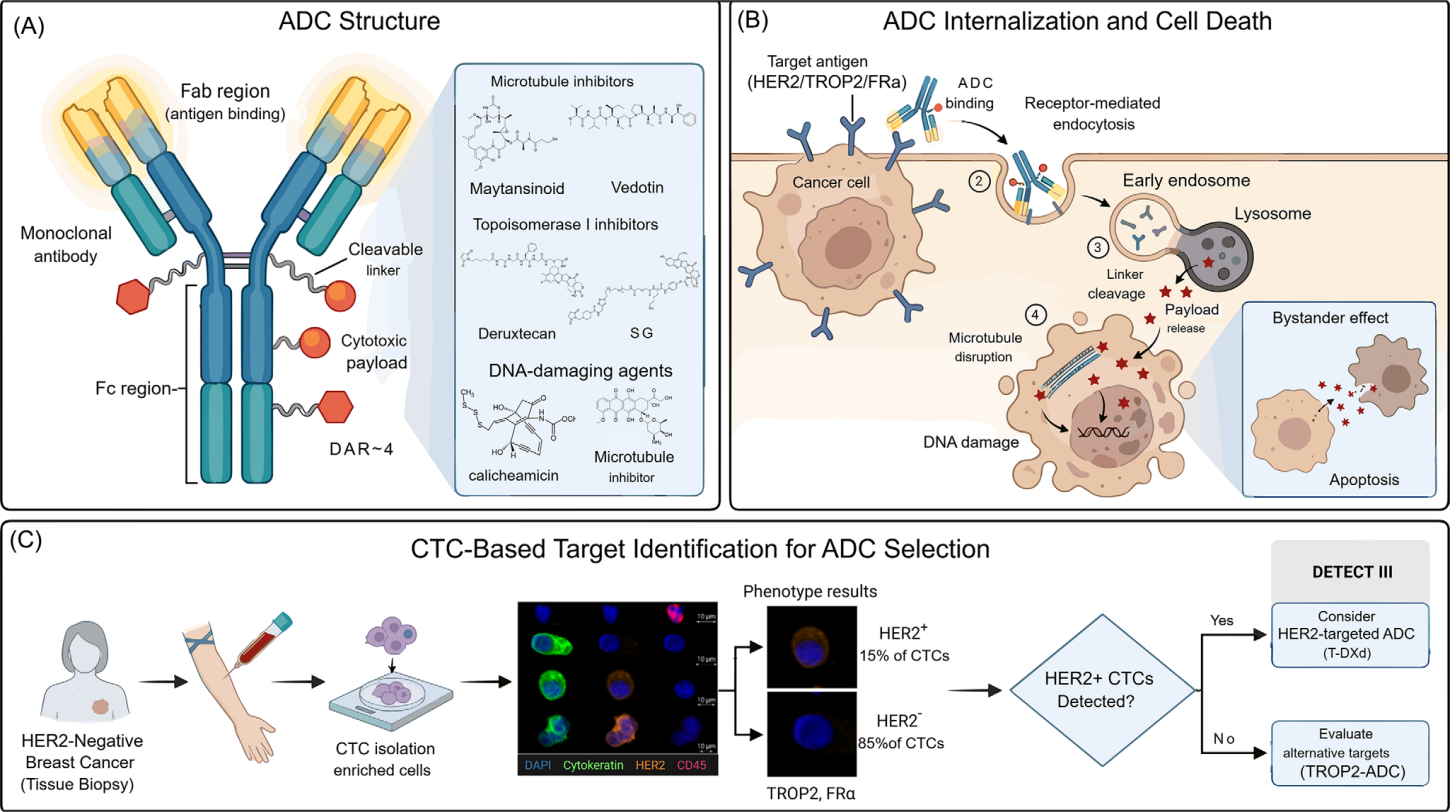
Antibody‒drug conjugate (ADC) mechanism of action and circulating tumour cell (CTC)‐based target identification. (A) Structure of antibody‒drug conjugates showing monoclonal antibody, cleavable linker and cytotoxic payload components with typical drug‐to‐antibody ratio of ∼4. Inset shows major payload classes. Chemical structures are indicative. (B) Mechanism of ADC action: target‒antigen binding, receptor‐mediated endocytosis, lysosomal processing with linker cleavage, payload release and induction of cell death, with bystander effect on adjacent cells. (C) Clinical workflow for CTC‐based ADC patient selection, demonstrating identification of HER2^+^ CTCs in tissue human epidermal growth factor receptor 2 (HER2)‐negative breast cancer patients, enabling consideration of HER2‐targeted ADC therapy based on liquid biopsy phenotyping.^36^ Created by FigureLabs.

ADCs consist of a tumour‐targeting monoclonal antibody, a chemical linker and a cytotoxic payload (Figure 2A). Current FDA‐approved agents employ payloads across three main classes: microtubule inhibitors, topoisomerase I inhibitors and DNA‐damaging agents (Table 1).^35^

**TABLE 1:** Food and Drug Administration (FDA)‐approved antibody‒drug conjugates (ADCs) and potential circulating tumour cell (CTC)‐based biomarker applications.

ADC (trade name)	Target	Indication(s)	FDA approval	Evidence level	Potential CTC‐based biomarker application	Ref.
Gemtuzumab ozogamicin (Mylotarg)	CD33	AML	2000/2017	Exploratory	CD33^+^ CTC detection; minimal residual disease monitoring	^37^
Brentuximab vedotin (Adcetris)	CD30	HL, ALCL	2011	Hypothetical	CD30^+^ circulating tumour cells in lymphoma	^38^
Trastuzumab emtansine (Kadcyla)	HER2	HER2^+^ breast cancer	2013	Exploratory	HER2^+^ CTC enumeration; CTC HER2 amplification status	^39^
Inotuzumab ozogamicin (Besponsa)	CD22	ALL	2017	Exploratory	CD22^+^ blast monitoring in peripheral blood	^40^
Polatuzumab vedotin (Polivy)	CD79b	DLBCL	2019	Hypothetical	Circulating lymphoma cell detection	^41^
Enfortumab vedotin (Padcev)	Nectin‐4	Urothelial carcinoma	2019	Hypothetical	Nectin‐4 expression on CTCs; EMT marker assessment	^42^
Trastuzumab deruxtecan (Enhertu)	HER2	Breast, gastric, NSCLC	2019	Hypothetical	HER2‐low CTC quantification; real‐time target monitoring	^43^
Sacituzumab govitecan (Trodelvy)	TROP2	TNBC, HR^+^/HER2‐ BC	2020	Exploratory	TROP2^+^ CTC enumeration; EMT phenotype assessment	^44^
Loncastuximab tesirine (Zynlonta)	CD19	DLBCL	2021	Hypothetical	CD19^+^ circulating cell monitoring	^45^
Tisotumab vedotin (Tivdak)	TF	Cervical cancer	2021	Hypothetical	TF expression on CTCs; coagulation marker correlation	^46^
Mirvetuximab soravtansine (Elahere)	FRα	Ovarian cancer	2022	Exploratory	Folate receptor α^+^ CTC detection	^47^
Datopotamab deruxtecan (Datroway)	TROP2	HR^+^/HER2‐ BC, NSCLC	2024	Hypothetical	TROP2 heterogeneity assessment on CTCs	^48^
Telisotuzumab vedotin (Emrelis)	c‐Met	NSCLC (c‐Met high)	2025	Hypothetical	c‐Met overexpression on CTCs; resistance monitoring	^49^

Abbreviations: ALCL, anaplastic large cell lymphoma; ALL, acute lymphoblastic leukaemia; AML, acute myeloid leukaemia; BC, breast cancer; DLBCL, diffuse large B‐cell lymphoma; EMT, epithelial‒mesenchymal transition; FRα, folate receptor alpha; HER2, human epidermal growth factor receptor 2; HL, Hodgkin lymphoma; HR, hormone receptor; mTNBC, metastatic triple‐negative breast cancer; NSCLC, non‐small cell lung cancer; TF, tissue factor; TROP2, trophoblast cell surface antigen 2.

Upon binding to target antigen‐expressing cells, the ADC‒receptor complex undergoes receptor‐mediated endocytosis, followed by lysosomal trafficking and proteolytic cleavage of the linker, releasing the cytotoxic payload intracellularly.^9^ Importantly, the efficacy of ADCs is not solely dictated by the presence of the target antigen, but also a function of a multistep pharmacological cascade involving antigen density (which influences stoichiometry of payload delivery), internalisation rate and lysosomal processing efficiency (which can vary by target‒antibody pairing independent of surface expression level), linker stability (which impacts premature payload release in circulation) and intracellular payload sensitivity (which is affected by factors such as drug efflux pump activity and the integrity of apoptotic pathways) that ultimately dictate whether antigen engagement is converted into cytotoxicity.^11^, ^13^, ^38^ Membrane‐permeable payloads can also diffuse into neighbouring cells regardless of their own target expression, a phenomenon called the ‘bystander effect’, which may enhance ADC efficacy in heterogeneously expressing tumours but also contributes to off‐target toxicity.^50^ In solid tumours, efficacy is further constrained by physical tumour penetration, as antibody‐sized molecules diffuse poorly beyond a few cell layers from the vasculature, particularly in densely stromal regions^14^ (Figure 2B).

Therefore, the clinical success of ADCs depends on sufficient and uniform expression of the target antigen. However, tumour heterogeneity, whether spatial or temporal, plays a significant role in ADC resistance, as it leads to notable variations in antigen expression across different tumour regions.^5^, ^15^ Even in tumours with overall high human epidermal growth factor receptor 2 (HER2) expression, as one of the ADC targets in breast cancer, significant HER2‐negative subpopulations may exist, which limit ADC penetration and coverage.^15^ In the ZEPHIR trial, approximately 46% of HER2‐positive patients had fewer than half their lesions showing uniform radiotracer uptake on HER2‐PET/CT, illustrating inter‐lesion heterogeneity of antigen distribution.^51^ Prolonged ADC treatment can further alter target expression (trastuzumab emtansine treatment significantly reduces HER2 protein levels in resistant cell lines), underscoring the value of dynamic, real‐time monitoring over reliance on a single archival biopsy.^5^, ^11^

### 2.1 What CTC antigen positivity can and cannot predict for ADC therapy

It is important to distinguish target antigen detection from ADC sensitivity prediction. CTC‐based antigen positivity reliably indicates target availability and captures spatial and temporal heterogeneity in expression across metastatic sites and over the treatment course.^52^, ^53^ However, antigen positivity alone does not predict antigen density at a level that would allow estimation of payload delivery, internalisation efficiency or trafficking kinetics, target‒antibody pair dependent and independent of expression level, intracellular payload sensitivity including efflux pump activity or impaired apoptotic signalling that may confer resistance despite robust antigen expression, or tumour penetration, since CTCs are sampled from the vascular compartment and do not reflect the stromal and physical barriers that limit ADC distribution within solid tumour deposits.^14^ CTC antigen positivity should therefore be interpreted as an indicator of biomarker eligibility rather than a surrogate for ADC sensitivity. Functional assays, such as CTC‐derived organoid (CTCDO) drug testing, may be needed alongside antigen profiling to more fully capture the determinants of ADC response.^11^, ^13^, ^15^

### 2.2 Circulating tumour cells as dynamic indicators of tumour heterogeneity

CTCs offer a new approach to solving the problem of tumour heterogeneity in ADC selection. In contrast to tissue biopsies which are informative from only one tumour site, CTCs are shed from multiple metastatic lesions into the bloodstream, giving a more complete picture of total tumour burden and its molecular diversity.^52^, ^53^ Single‐cell profiling of CTCs has shown marked heterogeneity in biomarker expression, including hormone receptors and HER2 as tumours evolve during the course of disease.^54^ Importantly, CTC analysis can detect phenotypic changes during therapy, providing insight into clonal evolution and resistant subpopulations.^55^ For example, greater EpCAM expression variation occurs across patient‐derived CTCs than in cell‐line controls, reflecting the heterogeneous nature of the metastatic process.^56^ This ability to characterise tumour heterogeneity makes CTC analysis a potentially valuable tool for stratifying patients receiving ADC therapy based on real‐time target‒antigen status, although its clinical utility in this specific context requires further prospective validation.

AI‐based image analysis is increasingly used to capture this heterogeneity more comprehensively than conventional marker‐based gating allows. Deep contrastive learning frameworks trained on whole‐slide immunofluorescence images have achieved over 92% accuracy in classifying diverse CTC phenotypes directly from single‐cell image features, without relying on predefined marker thresholds. This enables more complete characterisation of phenotypic diversity, including rare or atypical subpopulations that conventional gating strategies may miss.^57^ Such approaches could ultimately support more granular antigen‐heterogeneity mapping relevant to ADC target selection.

### 2.3 CTC phenotyping for patient stratification in ADC therapy

A primary use of CTC phenotyping is to find patients whose antigen status differs between primary and metastatic sites. Studies show that 10%–20% of patients with HER2‐negative primary breast cancer have HER2‐positive CTCs. These CTCs may respond to HER2‐targeted therapies such as ADCs.^16^, ^58^ The DETECT III trial was the first prospective phase III study to demonstrate that CTC phenotyping can identify HER2‐positive subclones in patients with tissue HER2‐negative breast cancer (Figure 2C).^16^ In this study, 2137 patients were screened for HER2‐positive CTCs, and eligible patients were randomised to receive lapatinib (a tyrosine kinase inhibitor) or conventional therapy, with overall survival (OS) considerably longer in the lapatinib arm (20.5 vs. 9.1 months, *p* = .009).^16^ Importantly, DETECT III provides indirect rather than direct support for CTC‐guided ADC selection. It establishes that HER2‐positive CTCs can identify a clinically actionable patient subset, but the intervention was not an ADC, and extrapolation of these findings to HER2‐targeted ADCs such as trastuzumab deruxtecan requires a dedicated prospective study.

The inclusion of a trial of a tyrosine kinase inhibitor in a review focused on ADCs warrants explicit mechanistic justification. Lapatinib acts entirely intracellularly: once it diffuses across the cell membrane, it reversibly occupies the cytoplasmic ATP‐binding pocket of the HER2 and epidermal growth factor receptor (EGFR) kinase domains, blocking receptor autophosphorylation and downstream PI3K/Akt and Ras/MAPK signalling.^59^ This mechanism requires no antibody‒antigen engagement, no receptor‐mediated endocytosis and no intracellular trafficking of the kind that determines ADC activity. ADCs, by contrast, depend on the multistep pharmacological cascade described above, extracellular antibody binding, receptor‐mediated endocytosis, lysosomal processing, linker cleavage and payload release, each step representing an independent point of failure that simply has no counterpart in lapatinib's mechanism.^9^, ^11^, ^14^ Therefore, DETECT III shows only that HER2 detection by CTC identifies a clinically actionable population that benefits from a HER2‐directed agent of any mechanistic class. It does not and cannot establish that the same population would respond to a HER2‐targeted ADC because antigen detection on CTCs provides no information about internalisation efficiency, lysosomal processing or intracellular payload sensitivity, determinants specific to ADC pharmacology and absent from small‐molecule tyrosine kinase inhibitor activity altogether. DETECT III thus provides evidence that HER2 detection based on CTC can identify a clinically actionable patient subgroup, but does not constitute evidence that this subgroup would derive comparable benefit specifically from a HER2‐targeted ADC.

AI‐assisted image classification is also being explored to standardise the kind of HER2‐status calls made in trials such as DETECT III, where manual immunofluorescence scoring is subject to inter‐observer variability. Automated, deep learning‐based classification pipelines have demonstrated high concordance with expert manual scoring while substantially reducing inter‐operator variability in CTC phenotype calls.^30^, ^60^ Applying such automated classification to HER2, baseline trophoblast cell surface antigen 2 (TROP2), or folate receptor alpha (FRα) phenotyping on CTCs could improve the reproducibility of antigen‐positivity calls used for ADC‐relevant patient stratification.

### 2.4 Beyond enumeration: molecular and multi‐omics profiling of CTCs

Beyond simple counting, detailed molecular analysis of CTCs improves how patients are selected for ADC therapy. Recent advances in single‐cell genomic profiling reveal resistant clones and ongoing microevolution that may affect treatment outcomes.^55^, ^61^ Single‐cell RNA sequencing (scRNA‐seq) and multi‐omics approaches have revealed heterogeneity within CTC populations and identified molecular signatures linked to therapeutic responses.^62^ TROP2‐positive CTCs correlate with a favourable response to sacituzumab govitecan (SG), and decreases in CTCs during treatment suggest therapeutic benefit.^51^ As a first‐in‐class ADC, SG targets TROP2 in metastatic triple‐negative breast cancer (mTNBC).^63^ Similarly, the absence of HER2 amplification detected by ctDNA analysis after trastuzumab deruxtecan therapy (Table 1) is associated with higher objective response rates than in patients with ongoing amplification.^64^ Integrating these molecular layers (protein, transcript and genomic) at single‐cell resolution generates high‐dimensional data that exceed what manual interpretation can practically synthesise, a complexity already evident in scRNA‐seq and multi‐omics studies of CTC heterogeneity discussed above.^62^ AI‐based multi‐omics integration frameworks have been proposed to combine antigen expression, transcriptomic and genomic CTC data into composite profiles intended to guide ADC target selection. At this point in the review, it is sufficient to note that molecular CTC profiling, the basis for such AI integration, is technically demanding and not yet standardised across laboratories, consistent with the broader lack of standardised protocols among CTC platforms discussed later in this review.

### 2.5 Dynamic target antigen monitoring using liquid biopsy

The dynamic expression of tumour markers supports the inclusion of CTC analysis in the therapeutic approaches of ADC. Recent studies have shown that HER2 status can change during disease progression and treatment and may affect eligibility for ADCs. For example, in TNBC, the proportion of patients with at least one HER2‐low result increased dramatically, from 59% after one biopsy to 100% after five or more. This suggests that a single biopsy could miss potential candidates for trastuzumab deruxtecan.^65^ Numerous studies have demonstrated significant discordance between tissue and CTC HER2 status, with a discordance rate of 32.6% reported between primary lesions and real‐time CTC profiles, potentially resulting in the inadequate treatment of HER2‐low or ‘HER2‐hidden’ populations.^66^ Furthermore, nearly 30% of breast cancers undergo HER2 status changes during neoadjuvant treatment.^67^ In gastric cancer, real‐time HER2 evaluation on CTCs has indicated that HER2 status might be a more accurate predictor of benefit from HER2‐targeted therapy than tissue biopsies, particularly in cases with heterogeneous expression.^68^

In ovarian cancer, ADCs such as mirvetuximab soravtansine, which targets FRα, are mostly used in recurrent or platinum‐resistant cases. However, biomarker testing is usually done on archival tissue from the initial surgery.^47^ Studies show significant heterogeneity in FRα expression: primary tumours have higher positivity rates (44.4%) compared to metastatic sites (32.5%), and 37.5% of patients show discordant FRα results between specimens.^69^ Additionally, FRα expression can change over time during disease progression, with up to 44% of patients showing discordance between primary and recurrent samples.^70^ In this context, tumour cells obtained from malignant ascites and pleural effusions, which are common in advanced ovarian cancer and highly enriched for cancer cells, offer a practical, complementary method for real‐time evaluation of ADC targets.^71^ Ascites‐derived tumour cells have shown molecular profiling accuracy similar to tissue biopsies and serve as an accessible source for biomarker analysis throughout the disease.^72^

B7‐H3, the target for ifinatamab deruxtecan (which recently received FDA Breakthrough Therapy Designation for extensive stage small cell lung cancer [SCLC]), is overexpressed in about two‐thirds of SCLC patients. However, early studies have not found a clear link between tissue expression levels and therapeutic response, indicating that dynamic monitoring might be needed to improve patient selection and treatment management.^73^ Likewise, Nectin‐4 expression in urothelial carcinoma exhibits profound changes throughout disease progression, with 59% of metastatic tumours having reduced expression in comparison to primary tumours, possibly resulting in lessened efficacy of enfortumab vedotin.^74^ Tissue factor (TF), the target of tisotumab vedotin in cervical cancer, and CDH6, the target of raludotatug deruxtecan in platinum‐resistant ovarian cancer, also demonstrate expression heterogeneity that may vary during treatment, although liquid biopsy approaches for these markers are still being explored.^75^, ^76^

These findings demonstrate how liquid biopsy approaches can identify patients who may benefit from ADC therapy despite initially negative or discordant tissue results, underscoring the clinical value of dynamic target monitoring across the expanding ADC therapeutic landscape.

### 2.6 Integrating CTCs with multimodal liquid biopsy strategies

A multimodal approach to optimise patient selection for ADCs can be achieved by combining CTCs with molecular imaging and ctDNA. HER2‐PET/CT imaging captures the total tumour burden at a given time, identifies heterogeneity at the lesion level, and identifies patients who are likely to benefit from anti‐HER2 ADCs.^51^, ^77^ When combined with CTC phenotyping and ctDNA analysis, clinicians can gain a comprehensive understanding of target antigen status across all disease sites while tracking molecular evolution over time.^53^, ^64^ This integrated approach is especially valuable given that clinical trials such as SURVIVE‐HERoes now evaluate trastuzumab deruxtecan in patients with HER2‐positive or HER2‐low early breast cancer who experience molecular relapse detected by ctDNA, marking a shift towards liquid biopsy‐guided therapy escalation.^78^

A similar model is emerging for prostate‐specific membrane antigen (PSMA)‐targeted therapies in prostate cancer, with PSMA‐PET/CT now the standard for whole‐body target assessment. A combination of PSMA‐PET and ctDNA provides distinct yet complementary prognostic information so that imaging reveals anatomical localisation, while ctDNA reflects the genomic profile of circulating disease.^79^ Likewise, CTC‐based PSMA detection with greater than 99% analytical accuracy offers a minimally invasive alternative to PSMA‐PET imaging, with particular value in regions with limited imaging access and for longitudinal monitoring during PSMA‐targeted ADC therapy.^80^ Multiple ADC targets are under investigation in ovarian cancer including FRα (mirvetuximab soravtansine), CDH6 (raludotatug deruxtecan), NaPi2b and TF. For instance, the development of FRα‐targeted immuno‐PET imaging agents demonstrated the potential for non‐invasive whole‐body target assessment to complement tissue‐based companion diagnostics.^81^

For targets where a distinct molecular imaging modality is not yet available, such as Nectin‐4 in urothelial cancer, B7‐H3 in SCLC, and TF in cervical cancer, the combination of CTC phenotyping with ctDNA analysis offers a practical strategy to assess target expression heterogeneity and monitor for the emergence of treatment resistance. As the ADC landscape grows to include bispecific antibodies and new targets such as CDH6 and B7‐H3, multimodal liquid biopsy methods that combine CTCs, ctDNA and molecular imaging would become increasingly vital for managing tumour heterogeneity, selecting appropriate patient groups, monitoring treatment effectiveness and optimising therapeutic sequencing within the ADC field.^10^, ^82^

## 3 CTCS IN THERAPY MONITORING AND PROGNOSIS

Numerous clinical studies have clearly confirmed the prognostic importance of CTCs. For example, a survey by Cristofanilli et al. showed that metastatic breast cancer patients with ≥5 CTCs per 7.5 mL of blood had significantly shorter progression‐free survival (PFS) and OS compared to those with fewer CTCs.^83^, ^84^ The findings of this study led researchers to propose stratifying stage IV disease into aggressive (≥5 CTCs) versus indolent (<5 CTCs) categories based on CTC thresholds, reflecting distinct biological behaviours.^85^ A meta‐analysis confirmed these findings, showing hazard ratios of 2.55, 4.36 and 5.20 at CTC thresholds of ≥1, ≥2 and ≥5, respectively, highlighting the dose‒response relationship between CTC load and clinical outcomes.^53^, ^86^

### 3.1 CTC counts as a tool for treatment selection and escalation

Beyond prognostication, CTC enumeration has been clinically useful in guiding treatment choices. The important STIC CTC trial randomly assigned 755 patients with hormone receptor‐positive, HER2‐negative metastatic breast cancer to either clinician‐driven or CTC count‐driven first‐line treatment selection.^87^ In the CTC‐guided group, patients with five or more CTCs were treated with chemotherapy, whereas those with fewer than five CTCs received endocrine therapy. Median PFS was 15.5 months in the CTC group and 13.9 months in the standard group, achieving the primary non‐inferiority endpoint of the trial. In the subgroup of patients with discordant results (clinically low risk but high CTC counts), patients who received chemotherapy based on their CTC results experienced significantly better outcomes with a lower risk of death than patients who only received endocrine therapy. These results are the first phase III evidence that CTC counts may be a meaningful biomarker to guide decisions regarding escalation of therapy.^87^

### 3.2 Longitudinal monitoring of CTCs during systemic therapy

The real‐time assessment of CTC levels during treatment offers additional prognostic information beyond initial measurements. The SWOG0500 trial examined whether early changes in chemotherapy, based on consistently high CTCs after one cycle, could lead to better clinical outcomes.^88^ Although the primary goal was not achieved, patients whose CTCs remained high (≥5) after treatment began had a median OS of only 13 months, indicating they might be resistant to chemotherapy and could benefit from alternative targeted therapies. The CirCe01 trial showed that patients on third‐line or later chemotherapy who did not achieve CTC clearance and altered their treatment regimen experienced better survival in subgroup analyses.^89^ Longitudinal monitoring of CTCs has proven to be superior to single‐time‐point assessments, with research indicating that consistently elevated or continuously rising CTC counts provide more reliable prognostic information than baseline enumeration alone.^90^

Computational approaches are beginning to refine how longitudinal CTC count data are interpreted. In a preprint analysis of CTC count dynamics in patients with lung, breast and nasopharyngeal cancers, an automated image‐classification pipeline (CTC‐Quant) found that short, defined treatment‐cycle windows of CTC count change more clearly discriminated responders from non‐responders than raw longitudinal counts or simple change‐from‐baseline measures, which fluctuated too erratically to be clinically interpretable on their own.^91^, ^92^ While this finding requires validation in larger, prospective, ADC‐specific cohorts before clinical application, it illustrates how AI‐assisted longitudinal modeling may help extract clinically meaningful trends from inherently noisy serial CTC data, a challenge directly relevant to using CTC kinetics for ADC response monitoring, discussed further in Section 5.

### 3.3 CTC clusters as highly metastatic and prognostically relevant entities

CTC clusters, composed of two or more tumour cells that have aggregated, exhibit a 20‒100‐fold higher metastatic potential than individual CTCs.^93^ These multicellular groups exhibit improved survival in circulation due to their collective resistance to shear stress and anoikis, and they are more efficient at colonising distant sites. Using the CellSearch platform, approximately 17%–20% of people with metastatic breast cancer have CTC clusters.^94^ The presence of CTC clusters independently predicts lower PFS and OS, providing prognostic value beyond just counting solitary CTCs. A preclinical study using a CTC‐derived cell‐line model established from a breast cancer xenograft identified a gene signature, including upregulation of SPARC, THBS1, VCL and HSP90AB1, associated with metastatic behaviour in this model, with expression of these genes also correlating with worse outcomes in public patient gene‐expression datasets.^95^ As this work derives from a preclinical model and has not yet undergone peer review, the proposed signature should be regarded as hypothesis generating rather than clinically validated, pending confirmation in direct analysis of patient‐derived CTC clusters.

### 3.4 CTCs and minimal residual disease detection in early‐stage disease

CTC detection supports minimal residual disease (MRD) monitoring in a number of solid tumours allowing identification of patients at higher risk of recurrence and informing precision intervention strategies. In Bidard's study of early‐stage breast cancer, baseline CTC count was a strong prognostic marker. When data were combined from 21 studies, the detection of ≥1 CTC before neoadjuvant chemotherapy was an independent predictor of shorter disease‐free and OS, and the detection of additional CTCs was associated with a progressively worse prognosis.^86^ In addition to CTC counting, the clinical value of CTCs for MRD detection extends to characterisation based on gene expression, DNA methylation and phenotypic analysis, which can identify potential disease up to 4 years before clinically detectable metastasis.^96^

In resected non‐small cell lung cancer (NSCLC), postoperative CTC detection has demonstrated robust prognostic significance. A prospective study by Bayarri et al. showed that CTC presence one month after surgery was an independent predictor of early recurrence (hazard ratio = 5.75, *p* = .01), with preoperative CTC positivity observed in over 50% of patients.^97^ A larger retrospective cohort of 347 stage I‒IIIA NSCLC patients confirmed that preoperative CTC concentration is an independent prognostic factor (hazard ratio = 5.49) and can be incorporated into nomograms for recurrence prediction.^98^

Combining CTC monitoring with ctDNA analysis offers more insights: CTCs provide cellular‐level phenotypic data, such as target antigen expression levels, while ctDNA provides genomic profiling of resistance mutations, such as ESR1 changes in endocrine‐resistant disease.^54^ Additionally, ctDNA‐based molecular residual disease (MRD) tests can detect molecular relapse 8–12 months before clinical signs appear.^99^ Recent trials such as c‐TRAK‐TN, PADA‐1 and SERENA‐6 have shown that using ctDNA to guide treatment selection improves PFS, laying the groundwork for precision intervention strategies that integrate both CTC phenotyping for target antigen assessment and ctDNA for genomic profiling to guide ADC therapy escalation or de‐escalation.^100^

### 3.5 CTC‐based monitoring to optimise ADC therapy and treatment sequencing

Integrating CTC‐based monitoring with ADC therapy is an area of research that is expanding. Evaluating target antigen expression on CTCs in real time during treatment can help detect early signs of acquired resistance, such as downregulation of the target antigen or phenotypic switching. New multivariate predictive systems are also emerging in this space; for example, the tissue‐based ADC treatment response score (ADC‐TRS), which combines target expression with proliferation and adhesion markers, has shown promising correlation with clinical benefit across multiple ADC‒tumour type combinations in retrospective analyses presented at recent conferences, although this work has not yet been peer‐reviewed or prospectively validated.^101^ While ADC‐TRS is itself a tissue‐based assay rather than a CTC‐based one, it illustrates the kind of multivariate, density‐aware scoring approach that could plausibly be adapted to a CTC context, an extension that, to our knowledge, has not yet been attempted. For patients undergoing sequential ADC therapies (a rising strategy), monitoring CTC dynamics can help determine the best time to switch treatments.^102^ Advances in multimodal liquid biopsy techniques, which combine CTC enumeration, phenotyping and ctDNA analysis with imaging methods such as HER2‐PET/CT, enable comprehensive evaluation of target‒antigen status. This multimodal approach holds promise for enabling more personalised ADC selection and continuous monitoring throughout therapy, although prospective evidence in ADC‐specific cohorts is needed to confirm clinical benefit.^103^

Looking beyond enumeration‐ and antigen‐based monitoring alone, AI‐guided decision support frameworks have been proposed to synthesise longitudinal CTC antigen trajectories with ctDNA and clinical variables to inform ADC treatment sequencing. For example, early loss of target antigen expression could be flagged as a potential trigger to switch ADCs before radiographic progression becomes apparent. As detailed in Section 3, no such system has yet been prospectively validated specifically in ADC‐treated patients, and current frameworks remain preclinical or conceptual.^8^, ^104^ We highlight this concept here to make explicit how AI‐based treatment‐sequencing logic would build directly on the antigen‐monitoring approaches just described, rather than treating AI as a separate, later‐arriving topic.

### 3.6 CTC‐guided patient stratification for ADC therapy: from detection to treatment selection

Implementing CTC analysis for ADC therapy requires a careful, step‐by‐step approach to navigate both technical challenges and clinical considerations. Detecting CTCs involves finding the right balance between sensitivity and practicality because these cells are quite rare. Usually, identifying at least five CTCs in 7.5 mL of blood is a key indicator for prognosis in patients with metastatic disease.^83^ The FDA‐approved CellSearch system remains the standard for enumeration, utilising EpCAM‐based immunomagnetic enrichment.^105^ However, this method might miss cells undergoing epithelial‒mesenchymal transition (EMT). To obtain a more diverse cell population, label‐free enrichment based on physical properties such as size and deformability is needed.^106^

Beyond simple enumeration, CTC phenotyping allows for more personalised matching to targeted therapies by developing specialised scoring systems (Table 1). One example is the circulating tumour cell‐endocrine therapy index (CTC‐ETI), which combines absolute CTC counts with a multi‐marker profile (including ER, Bcl‐2, HER2 and Ki67) to better predict treatment resistance in hormone receptor (HR^+^) metastatic breast cancer than count alone.^107^ ADC‐TRS that has been described earlier above is an example of the multi‐parametric, density‐aware scoring logic that could, in principle, be applied to CTC data, although no CTC‐based implementation has been reported to date. To date, ADC‐TRS has been presented only in conference abstracts, with biomarker‐positivity frequency correlating with published clinical trial response rates in a retrospective, observational cohort; prospective validation, peer‐reviewed publication, and a clearly defined cutoff determination methodology are not yet available.^101^

The DETECT III trial provided proof of concept that HER2‐positive CTCs can identify actionable subclones in tissue HER2‐negative patients. However, as the intervention was lapatinib rather than an ADC, this represents indirect evidence for CTC‐guided ADC therapy (Table 2).^16^ While the ≥5 CTCs/7.5 mL threshold remains the validated standard for prognosis, interventional trials have explored lower ‘positivity’ cutoffs to maximise patient eligibility for targeted agents. For instance, the DETECT III trial utilised the presence of even a single HER2‐positive CTC (defined by strong immunofluorescent staining) as an inclusion criterion for adding HER2‐targeted therapy to standard care.^108^ Expanding on this, DETECT IV and V demonstrated the feasibility of using CTC phenotypes to tailor complex regimens, such as combining endocrine therapy with targeted agents (e.g., Everolimus or Ribociclib) or comparing chemo‐combination strategies in patients with target‐positive CTCs.^109^, ^110^ Taken together, these trials demonstrated that targeting ‘hidden’ positive clones on CTCs could improve overall response rates and PFS in some subsets, even if the main tissue is negative. Nonetheless, although these clinical insights are important, the widespread implementation of these thresholds remains challenging; standardisation of the staining intensity and multi‐centre reproducibility of a single‐cell threshold are ongoing challenges that require rigorous quality control before such strategies can become a universal standard for ADC or targeted therapy selection.

**TABLE 2:** Clinical evidence grading for CTC guided ADC therapy.

Study	Design	Population	Treatment type	CTC assay	CTC cutoff	Sample size	Primary endpoint	Main clinical result	Evidence category	Evidence level
**(A) Prognostic CTC evidence**										
Cristofanilli et al. (2004)^83^	Prospective observational	MBC	Non‐interventional	CellSearch (EpCAM^+^)	≥5 CTCs/7.5 mL	177	PFS, OS	≥5 CTCs independently predicts shorter PFS and OS	Prognostic CTC evidence	Validated prognostic
SWOG S0500^88^	Phase III RCT	MBC on chemotherapy	Non‐ADC (chemotherapy switch)	CellSearch	≥5 CTCs/7.5 mL	595	OS	Early therapy switch based on CTC non‐clearance did not improve OS; confirmed CTC prognostic value	Prognostic CTC evidence	Validated prognostic
**(B) Predictive/interventional CTC‐guided therapy (non‐ADC)**										
STIC CTC^87^	Phase III RCT	HR^+^/HER2^−^ MBC	Non‐ADC (endocrine vs. chemotherapy)	CellSearch	≥5 CTCs/7.5 mL	755	PFS	CTC‐guided therapy selection non‐inferior to clinician driven; improved outcomes in discordant high‐CTC subgroup	Predictive/interventional (non‐ADC)	Validated predictive (non‐ADC)
DETECT III^16^	Phase III RCT	HER2^−^ MBC with HER2^+^ CTCs	Non‐ADC (lapatinib, TKI)	CellSearch + HER2‐IF	≥1 HER2^+^ CTC	2137 screened	OS	Lapatinib improved OS (20.5 vs. 9.1 months, *p* = .009); indirect support for CTC‐guided targeted therapy only (not ADC specific)	Predictive/interventional (non‐ADC)	Indirect/non‐ADC interventional
DETECT IV^110^	Phase II prospective	HR^+^/HER2^−^ MBC with ≥1 CTC	Non‐ADC (eribulin or everolimus + exemestane)	CellSearch	≥1 CTC/7.5 mL	Ongoing	PFS	CTC dynamics assessed during non‐ADC regimens; feasibility demonstrated	Predictive/interventional (non‐ADC)	Exploratory (non‐ADC)
TREAT‐CTC^111^	Phase II RCT	HER2^−^ EBC, CTC^+^ post‐neoadjuvant	Non‐ADC (trastuzumab, monoclonal antibody)	CellSearch	≥1 CTC/15 mL	206	CTC clearance	No significant CTC clearance difference with trastuzumab	Predictive/interventional (non‐ADC)	Negative interventional (non‐ADC)
**(C) ADC‐specific CTC‐guided therapy**										
CirCe T‐DM1^112^	Phase II single‐arm	HER2^−^ MBC with HER2^+^ CTCs	ADC (trastuzumab emtansine, T‐DM1)	CellSearch + HER2‐FISH	≥1 HER2^−^ amplified CTC	11 treated	ORR	1/11 PR; mPFS 4.8 months; limited proof‐of‐concept. target‐positive CTCs alone insufficient to predict ADC benefit	ADC‐specific CTC‐guided therapy	Limited proof‐of‐concept (ADC specific)
**(D) Tissue‐based ADC trials (CTC application proposed)**										
DESTINY‐Breast04^64^	Phase III RCT	HER2‐low MBC	ADC (trastuzumab deruxtecan, T‐DXd)	Tissue HER2 IHC/ISH (CTC monitoring proposed)	N/A (tissue‐based)	557	PFS, OS	T‐DXd significantly improved mPFS (10.1 vs. 5.4 months); CTC HER2 monitoring proposed as future companion diagnostic	Tissue‐based ADC trial (CTC proposed)	Hypothetical CTC application
DAISY^113^	Phase II	MBC across HER2 spectrum	ADC (trastuzumab deruxtecan, T‐DXd)	Tissue HER2 + ctDNA (CTC proposed)	N/A	220	ORR	65% HER2 decrease post‐progression; CTC HER2 monitoring proposed for real‐time tracking	Tissue‐based ADC trial (CTC proposed)	Hypothetical CTC application

Abbreviations: EBC, early breast cancer; HER2, human epidermal growth factor receptor 2; HER2‐FISH, HER2 fluorescence in situ hybridisation; HER2‐IF, HER2 immunofluorescence; HR, hormone receptor; IHC, mmunohistochemistry; ISH, in situ hibridization; MBC, metastatic breast cancer; mPFS, median progression‐free survival; N/A, not applicable; ORR, objective response rate; OS, overall survival; PFS, progression‐free survival; PR, partial response; RCT, randomised controlled trial; TKI, tyrosine kinase inhibitor.

## 4 ROLE OF ARTIFICIAL INTELLIGENCE AND MACHINE LEARNING

The automation of image‐based classification, standardisation of procedures and use of high throughput counting are applications that have obtained clinical‐grade validation and advanced CTC detection meaningfully using AI and ML.^29^ Traditional CTC identification involves manually examining immunofluorescence images, a process that is time consuming and subject to inter‐observer variability.^114^ Deep learning approaches, especially convolutional neural networks, have demonstrated high accuracy in automatically classifying CTCs from immunofluorescence in situ hybridisation (imFISH) images, achieving performance comparable to expert human reviewers and reducing the role of subjective assessment.^30^, ^60^ These algorithms distinguish CTCs from leukocytes and debris by analysing features such as nuclear size, cytoplasmic characteristics and fluorescence intensity patterns.^115^ Advanced architectures with attention mechanisms and transfer learning have improved classification accuracy across various CTC populations and have helped reduce, although not fully resolve, platform‐specific variability that has traditionally complicated data comparison between institutions.^116^ Integrating ML with microfluidic technologies enables real‐time data processing and pattern recognition, significantly increasing the speed and accuracy of CTC detection for clinical applications.

### 4.1 AI‐based integration of multi‐omics CTC data for ADC target selection

**FIGURE 3:**
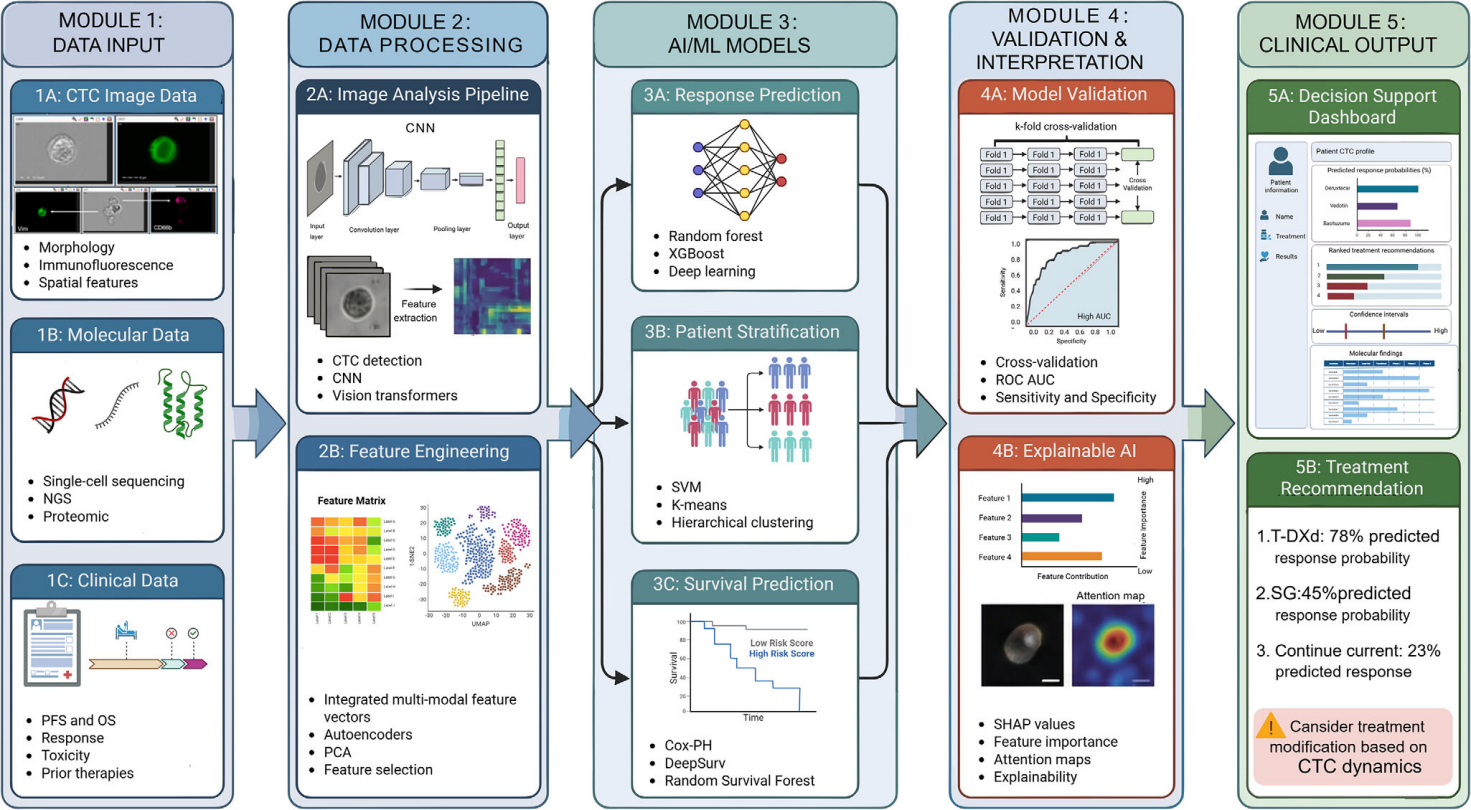
Artificial intelligence (AI) and machine learning (ML) pipeline for optimising circulating tumour cell (CTC)‐based antibody‒drug conjugate (ADC) therapy. Module 1: multimodal data input that includes CTC imaging (morphology and immunofluorescence), molecular profiling (single‐cell sequencing and proteomics) and clinical outcomes (response and survival). Module 2: data processing by analysing images (convolutional neural network [CNN] and U‐Net) and feature engineering (dimensionality reduction and multimodal integration). Module 3: AI/ML models for response prediction (Random Forest, XGBoost and Deep Learning), grouping patients (clustering) and survival analysis (DeepSurv). Module 4: model validation using cross‐validation and ROC analysis, along with explainable AI (SHAP values) for biological interpretation. Module 5: a clinical decision support system that offers ranked ADC recommendations predicted response probabilities and confidence intervals. Created by FigureLabs.

AI frameworks have been proposed to facilitate the integration of multi‐omics CTC data to guide ADC target selection and therapy optimisation. However, these applications remain exploratory and have not yet been validated in prospective clinical studies of ADC‐treated patients.^104^ scRNA‐seq, combined with ML classifiers, can precisely evaluate CTCs at the molecular level. Tree‐based algorithms trained on transcriptomic profiles can distinguish tumour cells from peripheral blood mononuclear cells with high sensitivity and specificity.^117^ Graph neural networks and transformer‐based models have been proposed to analyse complex datasets comprising antigen expression levels, genomic alterations and phenotypic features to rank ADC targets by tumour selectivity and internalisation. These approaches are currently preclinical and require prospective validation (Figure 3).^104^ In TNBC, computational analysis has shown that TROP2 is highly expressed on CTCs and is associated with EMT phenotypes, while this offers a plausible biological rationale for SG activity in aggressive disease subtypes, TROP2 expression on CTCs reflects target availability rather than confirmed payload sensitivity, and direct evidence linking CTC TROP2 levels to ADC response in this setting remains limited.^118^ These AI‐based approaches are promising in terms of measuring heterogeneity of CTC populations and in guiding ADC selection. However, their clinical utility over conventional immunohistochemistry of bulk tissue remains to be established in prospective trials.

A practical question raised by the data limitations discussed above is what sample size would be sufficient to train a clinically useful multi‐omics model for ADC response prediction. Using established minimum‐sample‐size frameworks for clinical prediction model development, a model with a modest number of candidate predictors (for example, 5–10 multi‐omics‐derived features) would, as a rough heuristic, require on the order of 10–20 response events per predictor parameter to limit overfitting, translating to a minimum of approximately 50–100 evaluable patients with a binary response outcome for a genuinely pilot scale, hypothesis‐generating model, assuming a roughly balanced response/non‐response distribution.^119^ This is broadly consistent with the scale of existing CTC‐based AI pilot studies discussed throughout this review, which have typically used cohorts of 30–80 patients per cancer type. Such pilot‐scale models should be considered exploratory and would require external validation in an independent cohort of comparable or larger size before any claim of clinical utility can be made. Scaling to a model intended for prospective clinical deployment, particularly one incorporating a larger number of multi‐omics features or requiring subgroup‐level reliability, would likely require cohorts in the many hundreds to low thousands of patients, consistent with the scale of tissue‐based multivariate biomarker discovery efforts in this space (e.g., the >15 000‐sample discovery cohort underlying the ADC‐TRS biomarker discussed earlier in this review). Until such CTC‐based multi‐omics datasets of this scale become available, pragmatic pilot studies in the 50–100 patient range with pre‐specified plans for external validation represent a realistic and statistically defensible near‐term target.

### 4.2 Predictive modelling of ADC response using longitudinal CTC and ctDNA data

Predictive ML models that combine CTC dynamics with ctDNA analysis have shown early promise for predicting outcomes and resistance to ADC therapy; however, these are still proof‐of‐principle studies lacking prospective clinical validation in cohorts treated with ADCs.^120^ In a prospective CTC imaging study of patients receiving TROP2‐ or HER2‐targeting ADCs, a marked decline in CTC numbers within three weeks of treatment initiation was strongly associated with durable response (TROP2: hazard ratio = 5.15, *p* = .012; HER2: hazard ratio = 6.01, *p* < .001).^36^ The same study found that target epitope expression on CTCs was not commonly downregulated at the time of acquired clinical resistance, and that switching between TROP2‐ and HER2‐targeting ADCs sharing similar payloads infrequently produced a second‐line response, pointing towards payload sensitivity, rather than epitope loss, as a primary driver of clinical response. This CTC‐based finding contrasts with a tissue‐based study of paired pre‐ and post‐treatment biopsies from 102 patients treated with trastuzumab deruxtecan, which found that 49% had a major decrease in HER2 expression at progression, with complete HER2 loss in just over half of those cases, and which demonstrated mechanistically that reduced HER2 expression directly impairs ADC internalisation and cytotoxicity.^121^ One way to reconcile these findings is a context‐dependent model in which target downregulation is more prominent in HER2‐low, non‐oncogene‐driven disease, whereas payload‐related resistance or rare binding‐site mutations predominate in HER2‐amplified, oncogene‐addicted tumours. Although this remains a hypothesis requiring further validation, and the discrepancy may also partly reflect differences between CTC‐based and tissue‐based antigen assessment. For TROP2, the peer‐reviewed final analysis of the TROPiCS‐02 trial similarly found no clear association between tissue TROP2 expression and SG benefit, consistent with the CTC‐based finding that target loss is a less dominant mechanism of resistance for TROP2‐directed ADCs specifically.^122^ Overall, whether epitope downregulation is a common resistance mechanism for ADC therapy appears to be both target‐ and tissue‐compartment‐dependent, and reconciling CTC‐ and tissue‐based observations remains an open question. ML frameworks that combine CTC counting with genomic features from ctDNA, such as changes in the RTK and ER pathways, have shown that they can predict therapy outcomes with more accuracy. This enables more precise risk stratification in metastatic breast cancer.^120^ The CirCe T‐DM1 trial provided limited proof‐of‐concept for CTC‐guided ADC therapy by identifying HER2‐amplified CTCs in HER2‐negative metastatic breast cancer patients via FISH analysis; however, clinical activity was modest, only one partial response among eleven treated patients with a median PFS of 4.8 months, suggesting that target‐positive CTC detection alone is insufficient to predict ADC benefit and that this study should not be interpreted as clinical validation of the approach.^112^

### 4.3 AI‐guided clinical decision support and ADC treatment optimisation

AI‐powered clinical decision support systems have been proposed to convert CTC biomarker data into treatment recommendations for ADC therapy, while none of these systems has yet been prospectively validated in ADC‐treated patients.^8^, ^104^ Preclinical platforms that incorporate real‐time enumeration of CTCs, target antigen measurement and molecular profiling have been described, but their clinical implementation for ADC selection, dosage adjustment and treatment sequencing remains theoretical. The ADC‐TRS exemplifies this conceptual approach, using target expression levels, proliferation and adhesion markers to predict response to a specific ADC agent, although, as noted above, this remains preliminary, tissue‐based, conference‐presented work rather than a CTC‐based or peer‐reviewed clinical decision‐support tool.^101^ Digital twin models and adaptive dosing algorithms have been proposed to simulate ADC pharmacokinetics using patient‐specific CTC features, but none of these platforms have been validated for ADC use; therefore, this approach should currently be regarded as speculative.^104^ When resistance to treatment develops, AI analysis of CTC molecular changes can assist in designing rational sequencing strategies. Notably, switching ADC epitope targets while keeping a similar payload class has shown limited success in a CTC‐based clinical study, supporting the hypothesis that sequential ADC therapy may be more effective when different payload mechanisms are used rather than simply targeting different antigens.^36^ Additionally, natural language processing tools that analyse electronic health records and adverse event databases improve pharmacovigilance by identifying new safety signals in patients treated with ADCs.^104^

### 4.4 Challenges and future directions for AI‐guided CTC analysis in ADC therapy

Despite notable progress, several challenges must be addressed before AI‐guided CTC analysis can reach its full clinical potential in ADC therapy. A lack of data continues to be a significant challenge, especially for ADC‐specific training datasets that include conjugation effects, intracellular trafficking and clinical pharmacokinetics and pharmacodynamics.^104^ For regulatory approval and clinical use, models must be easy to interpret. Explainable AI architectures that identify specific molecular features influencing predictions help build trust among clinicians and enhance their understanding of how things work.^123^ The development of closed‐loop experimental platforms that combine AI predictions with high‐throughput validation could help accelerate the cycle of designing, building, testing and learning to optimise ADCs, although such platforms remain at an early conceptual stage for ADC applications. Multimodal foundation models trained on multiple data types such as imaging, genomics and clinical outcomes could go a long way to personalised ADC selection, although clinical translation would need dedicated prospective studies. However, as these technologies mature, the integration of AI‐driven CTC analysis with liquid biopsy‐guided precision oncology may gradually transition ADC therapy from empirical treatment selection to more personalised strategies based on real‐time tumour biology; accomplishing this vision will require rigorous prospective validation and regulatory acceptance.^29^, ^104^

## 5 CHALLENGES AND FUTURE DIRECTIONS

Although the technological advances in CTC‐based liquid biopsy have been considerable, the direct combination with ADC therapy is still in its infancy, mainly preclinical and proof‐of‐concept studies, and many hurdles are to be surpassed for this approach to fully reach its potential in precision oncology. To translate promising research into widespread clinical use, issues of biological complexity, standardisation of technology and regulatory approval must be addressed.^124^, ^125^ This section discusses these key issues, as well as new strategies and possible future directions for developing CTC‐guided ADC therapy.

### 5.1 Standardisation and clinical validation

The absence of standardised protocols among CTC detection platforms presents a significant obstacle to their clinical use and the comparison of data between different institutions.^21^, ^126^ The current methods for counting CTCs vary in capture efficiency, ranging from 42% to over 90%, depending on the technology employed.^126^ The CellSearch system remains the only FDA‐approved method for counting CTCs in prognostic tests for metastatic breast, prostate and colorectal cancers. However, it struggles to capturing CTC populations with diverse phenotypes.^105^, ^127^ Additionally, pre‐analytical factors such as the type of blood collection tube, timing of processing, storage conditions and sample handling can cause variability that affects result interpretation.^128^ Developing consensus guidelines for sample collection, processing, quality control and reporting is essential to support multi‐centre studies and secure regulatory approval.^129^

Beyond enumeration, clinical implementation of CTC‐guided ADC selection imposes additional, more stringent requirements. Antigen quantification on CTCs is currently performed by semi‐quantitative immunofluorescence intensity scoring, which is not sufficiently reproducible to support treatment‐defining decisions; for the implementation of ADC‐specific assays, quantitative, calibrated antigen assays will be needed, similar to standardised HER2 immunohistochemistry scoring in tissue. To implement ADCs clinically, reproducible staining thresholds and inter‐laboratory concordance studies are needed, which are currently lacking for ADC‐relevant targets such as HER2‐low, TROP2 and FRα on CTCs. Even small differences in threshold definition can lead to reclassification of patients as eligible or ineligible for a given ADC. Clear, target‐specific positivity cutoffs analogous to the validated ≥5 CTCs/7.5 mL prognostic threshold do not yet exist for ADC‐relevant antigens, and ad hoc thresholds, such as the single‐cell positivity criterion used in DETECT III, have not been validated against ADC clinical outcomes.^16^ Emerging multivariate scoring systems such as ADC‐TRS illustrate one possible direction for quantitative, density‐aware antigen assessment, combining target expression with proliferation and adhesion signatures.^101^, ^130^ Without reliable antigen quantification, reproducible thresholds, inter‐laboratory concordance and validated cutoff definitions, CTC‐based ADC selection cannot be implemented in routine clinical decision making.

**FIGURE 4:**
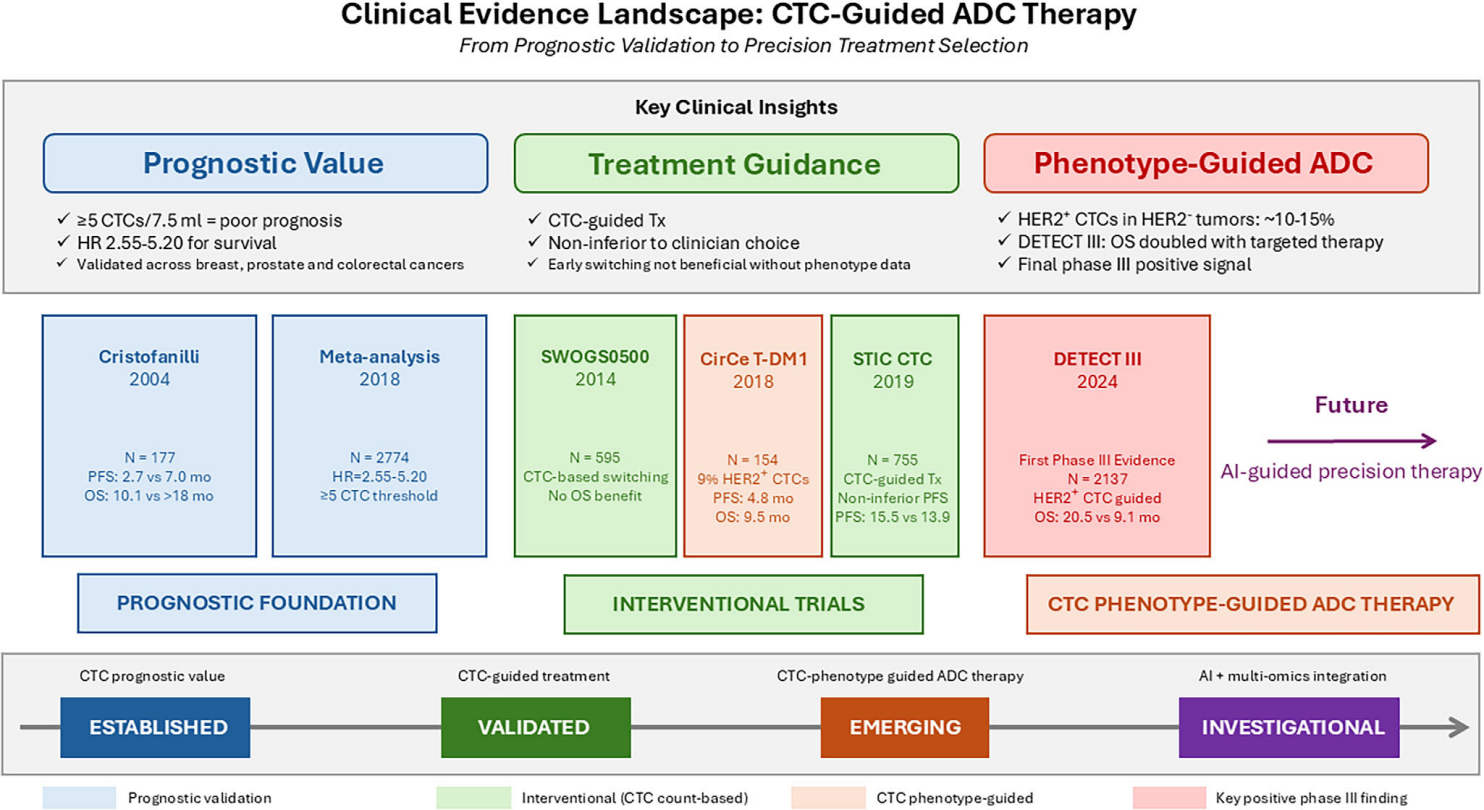
Clinical evidence landscape for circulating tumour cell (CTC)‐guided antibody‒drug conjugate (ADC) therapy. The timeline shows how clinical evidence has changed over time. It starts with prognostic validation (2004‒2018, blue), moves on to interventional trials (2014‒2019, green), and ends with CTC‐phenotype‐guided ADC therapy (2018‒2024, orange/red). The Cristofanilli study set the ≥5 CTCs/7.5 mL prognostic threshold, the STIC CTC study showed that CTC‐guided treatment selection is possible, and the DETECT III trial was the first phase III study to show that HER2^+^ CTC‐guided targeted therapy (with lapatinib, a tyrosine kinase inhibitor [TKI]) improves overall survival (20.5 vs. 9.1 months, *p* = .009) in human epidermal growth factor receptor 2 (HER2)‐negative metastatic breast cancer, representing indirect evidence for CTC‐guided ADC selection. The key clinical insights panel shows what was found in different stages, and the evidence maturity spectrum shows how the move from established prognostic value to artificial intelligence (AI)‐integrated approaches is happening.

Future clinical trials should include prospective CTC monitoring with standardised assays, establish clear thresholds for actionable target antigen expression, and compare the success of CTC‐guided treatments to standard care (Figure 4; see Table 2 for evidence grading). Collaborative efforts among academic centres, regulatory agencies and industry can speed up standardisation of CTC methodologies and provide the evidence needed to incorporate them into clinical guidelines.^131^, ^132^

### 5.2 Preserving CTC viability for functional studies

Maintaining CTC viability during isolation is essential so they can be used in later tests, such as ex vivo drug sensitivity assays and the development of patient‐derived models to predict ADC responses.^133^, ^134^ However, many enrichment techniques require fixation, immunomagnetic labelling or mechanical forces that damage cell integrity, limiting their use in functional studies.^135^ The overall success rate for culturing isolated CTCs remains low (<20%) because these cells do not survive well outside the body, it is difficult to mimic the TME, and circulating cells are fragile when exposed to haemodynamic stress.^136^ Label‐free microfluidic systems that use physical traits such as size and deformability (which include the Parsortix system, ClearCell FX1 and inertial focusing devices) are promising options that preserve cell viability while capturing cells without relying on markers.^137^, ^138^, ^139^ The versatility of these platforms extends beyond oncology: label‐free microfluidic devices have been widely applied to isolate healthy, motile sperm for assisted reproduction,^140^, ^141^, ^142^ and inertial‐focusing principles have even been repurposed to isolate CTCs directly from the seminal fluid of prostate cancer patients, underscoring how a single class of technology can gently separate viable target cells from complex biological fluids across both reproductive medicine and cancer diagnostics.^143^ Additionally, binary colloidal crystal substrates and specialised culture matrices have successfully expanded viable CTCs from SCLC patients, with drug sensitivity results correlating with clinical responses.^144^

CTCDOs have been proposed as a promising, but still early stage, approach for personalised ADC testing by linking liquid biopsy with functional oncology.^145^, ^146^ Given that baseline CTC culture success is already low (<20%, as noted above) and that organoid formation requires sustained proliferative expansion well beyond short‐term viability, CTCDO establishment rates are expected to be at least as limited, and the current literature does not report success rates approaching routine feasibility. These three‐dimensional models, when successfully established, have been reported to retain features of tumour heterogeneity, cellular interactions and microenvironmental characteristics that may influence ADC penetration, internalisation and payload release, although the consistency of this recapitulation across patient samples and cancer types has not been systematically validated.^147^ The need to actively modify pathways such as TGF‐β inhibition and p38‐MAPK activation simply to awaken dormant CTCs for culture further reflects the technical fragility of this approach.^145^ Biobanking efforts to assemble collections of well‐characterised CTCDO models for systematic ADC screening are underway, but low and variable success rates across cancer types currently limit the scale and representativeness of these biobanks. Combining CTCDO platforms with high‐throughput drug testing and AI‐driven response prediction could, in principle, support more personalised ADC decisions based on individual patient CTC biology; however, this remains a long‐term aspiration, and substantial improvements in culture efficiency and reproducibility are needed before near‐term clinical implementation should be expected.

### 5.3 Capturing phenotypically heterogeneous CTC populations

The EMT poses a major obstacle for EpCAM‐dependent CTC detection methods, potentially leading to underestimating the disease burden and missing clinically aggressive CTC subpopulations.^148^, ^149^ During EMT, epithelial markers such as EpCAM and cytokeratins are reduced. In contrast, mesenchymal markers such as N‐cadherin, vimentin and Twist are upregulated, thereby enabling CTCs to evade detection by standard techniques.^150^, ^151^ Comparative isolation studies have demonstrated that EpCAM‐based capture approaches yield a significantly lower percentage of CTCs than marker‐independent methods, particularly for cell populations with reduced expression of epithelial markers. In one direct comparison, EpCAM‐based immunomagnetic selection resulted in about 52% recovery versus up to 95% for marker‐independent filtration‐based isolation.^152^ This gap is expected to widen further for genuinely mesenchymal‐shifted CTC subpopulations not well represented in standard cell‐line spiking experiments. This is especially relevant for ADC therapy because EMT‐associated CTCs may have unique target expression profiles and could be treatment‐resistant populations that drive disease progression.^118^ In advanced gastric cancer, the ratio of mesenchymal (vimentin‐positive) to epithelial (EpCAM‐positive) CTCs showed significant prognostic value, with 96% of patients having M/E ratios above 1 experiencing disease progression.^153^

Several approaches are being investigated to facilitate the identification of cells that are involved in EMT. The use of multi‐marker capture cocktails with both epithelial (EpCAM) and mesenchymal (N‐cadherin and vimentin) antibodies has resulted in nearly a threefold increase in the CTC recovery rate in ovarian cancer patients, a significant improvement over the use of EpCAM alone.^154^ Cell surface vimentin may also function as a valuable marker for detecting EMT‐CTCs, as it can help identify more aggressive subpopulations that other methods might miss.^153^ Furthermore, CTCs can be isolated without the use of surface markers using label‐free microfluidic techniques that rely on size, deformability and dielectrophoretic properties.^139^ These methods achieve capture efficiencies between 70% and 99%, enabling detailed molecular analysis.^155^, ^156^ Finally, negative selection methods, which remove CD45‐positive leukocytes, offer alternative approaches to isolate phenotypically diverse CTC populations, which is crucial for ADC target evaluation and increasing tumour cell yield.^123^

These limitations raise a practical question relevant to the clinical interpretation of CTC‐based ADC biomarker data: should EpCAM‐based platforms such as CellSearch be abandoned in favour of marker‐independent methods? Based on the evidence reviewed here, we do not see a basis for recommending wholesale replacement of EpCAM‐based platforms at this time. CellSearch remains the only FDA‐cleared CTC enumeration platform and underlies the prognostic and predictive thresholds established in several of the major trials discussed in this review, including the ≥5 CTCs/7.5 mL cutoff from Cristofanilli et al., the STIC CTC and SWOG S0500 trials, and DETECT III. Despite higher recovery rates observed with marker‐independent and multi‐marker approaches in comparative studies, this review has not performed a systematic comparison of all available isolation platforms and the relative clinical utility of these approaches for ADC‐specific applications remains an open area of investigation and not a settled question.

A more cautious, practical conclusion is that EpCAM‐based and marker‐independent approaches likely complement each other rather than compete. The best choice depends on the clinical setting. In cases where EMT‐induced phenotypic changes are biologically plausible, such as heavily pretreated patients or tumour types with documented mesenchymal plasticity, marker‐independent methods in addition to EpCAM‐based assessments may be beneficial. For clinicians, the practical implication is that a negative or low EpCAM‐based CTC result should not, on its own, be interpreted as definitive evidence that an ADC‐relevant target is absent, since EpCAM‐low or EMT‐shifted tumour cells may be under‐represented by this method. Where clinical suspicion remains high despite a negative or low EpCAM‐based CTC count, complementary assessment, via marker‐independent CTC capture, ctDNA analysis, or repeat tissue biopsy, where feasible, may be a reasonable consideration, although this approach has not been formally validated and should be guided by clinical judgement.

### 5.4 Emerging technologies and future directions

scRNA‐seq of CTCs can be used for high‐resolution characterisation of tumour heterogeneity, EMT dynamics and ADC target expression at the single‐cell level, which is a powerful approach.^157^, ^158^ scRNA‐seq research has identified various CTC subtypes with distinct metastatic abilities, highlighting new therapeutic targets, including stress response pathways, immune evasion strategies and chemoresistance markers.^159^, ^160^ In ADC applications, single‐cell transcriptomics enable quantitative assessment of target‒antigen heterogeneity, identification of resistance‐associated transcriptional programs, and discovery of alternative targets within individual patient CTC populations.^161^ Combining spatial transcriptomics with CTC analysis can reveal intercellular interactions within CTC clusters and their influence on ADC efficacy.^162^

CTC‐guided precision oncology's next frontier will be defined by multi‐omics integration, combining genomic, transcriptomic, proteomic and epigenomic CTC profiling with AI‐driven analytics.^104^, ^163^ Comprehensive CTC datasets could, in principle, be used to train ML models aimed at predicting ADC response, informing treatment selection and flagging emerging resistance mechanisms; however, such models do not yet exist for ADC applications and would require substantial prospective validation.^120^ Clinical application of advanced characterisation techniques is now supported by the Parsortix system and comparable platforms, which facilitate workflow integration from CTC capture through single‐cell molecular analysis.^164^ With FDA approvals of ctDNA‐based tests establishing the standard for CTC‐based applications, regulatory processes are evolving to support liquid biopsy companion diagnostics.^165^

Emerging spatial profiling technologies may further complement CTC analysis by mapping ligand‒receptor interactions within the TME to refine ADC‒immunotherapy combination strategies.^166^

In the future, ADC therapy may gradually shift from static, biopsy‐defined protocols towards more dynamic, liquid biopsy‐guided approaches, contingent on the future development and validation of closed‐loop systems incorporating real‐time CTC monitoring, AI‐guided interpretation and adaptive treatment algorithms.^3^, ^167^

In summary, combining ADC therapy with CTC analysis, used as part of a complementary, multimodal liquid biopsy strategy alongside ctDNA and tissue‐based testing, rather than as a standalone or necessarily leading approach, represents a promising move towards more personalised cancer treatment, contingent on prospective clinical validation. Continuous developments in technologies such as microfluidics, nanotechnology and AI are slowly aiding in overcoming many of the challenges that remain, including standardisation, maintaining viability, capturing phenotypes and clinical validation. Collaboration between researchers, clinicians and industry is essential for successful implementation of CTC‐based biomarkers in the planning of ADC treatment. With the evolution of these tools, real‐time, tumour biology‐based customisation of therapy may improve outcomes.

## AUTHOR CONTRIBUTIONS

*Concept and design*: Vahid Yaghoubi Naei and Majid Ebrahimi Warkiani. *Literature review and figure preparation*: Vahid Yaghoubi Naei, Mehran Dabiri and I‐Han Wang. *Critical review and writing*: all the authors.

## CONFLICT OF INTEREST STATEMENT

The authors declare they have no conflicts of interest.

## ETHICS STATEMENT

Not applicable.
